# Functional annotation of putative QTL associated with black tea quality and drought tolerance traits

**DOI:** 10.1038/s41598-018-37688-z

**Published:** 2019-02-06

**Authors:** Robert. K. Koech, Pelly M. Malebe, Christopher Nyarukowa, Richard Mose, Samson M. Kamunya, Fourie Joubert, Zeno Apostolides

**Affiliations:** 1 Department of Biochemistry, Genetics and Microbiology, University of Pretoria, Pretoria, 0002 South Africa; 2 Kenya Agriculture and Livestock Research Organization, Tea Research Institute, P.O. Box 820, Kericho, 20200 Kenya; 3 James Finlay (Kenya) Limited, P.O. Box 223, Kericho, 20200 Kenya

## Abstract

The understanding of black tea quality and percent relative water content (%RWC) traits in tea (*Camellia sinensis*) by a quantitative trait loci (QTL) approach can be useful in elucidation and identification of candidate genes underlying the QTL which has remained to be difficult. The objective of the study was to identify putative QTL controlling black tea quality and percent relative water traits in two tea populations and their F1 progeny. A total of 1,421 DArTseq markers derived from the linkage map identified 53 DArTseq markers to be linked to black tea quality and %RWC. All 53 DArTseq markers with unique best hits were identified in the tea genome. A total of 5,592 unigenes were assigned gene ontology (GO) terms, 56% comprised biological processes, cellular component (29%) and molecular functions (15%), respectively. A total of 84 unigenes in 15 LGs were assigned to 25 different Kyoto Encyclopedia of Genes and Genomes (KEGG) database pathways based on categories of secondary metabolite biosynthesis. The three major enzymes identified were transferases (38.9%), hydrolases (29%) and oxidoreductases (18.3%). The putative candidate proteins identified were involved in flavonoid biosynthesis, alkaloid biosynthesis, ATPase family proteins related to abiotic/biotic stress response. The functional annotation of putative QTL identified in this current study will shed more light on the proteins associated with caffeine and catechins biosynthesis and % RWC. This study may help breeders in selection of parents with desirable DArTseq markers for development of new tea cultivars with desirable traits.

## Introduction

The identification of putative candidate genes underlying QTLs in plants is often complicated and time-consuming. This is even further complicated by genotype-environment interactions, which influence the growth and development of plants. However, using recently unveiled tea genome sequence^1^, it was possible to annotate candidate genes and enzymes involved in the biosynthesis of phenolic compounds and drought tolerance. The phenolic compounds in tea are closely associated to the classical flavour of tea infusions and to the health benefits ascribed to tea consumption^2,3^. Catechins (flavan-3-ols), flavonols, and their derivatives (galloylated catechins and flavonol 3-O-glycosides) are among the major biomolecules in tea with important bioactivities. The polyphenols in tea have been shown to contribute to the astringency in black tea infusions^4^. Especially, galloylated catechins have been found to confer astringent and bitter tastes in tea^5^ while flavonol 3-O-glycosides have been found to confer velvety, mouth-drying, and mouth-coating sensations^6^. During the production of black tea, the enzymatic oxidation of the leaf catechins by polyphenol oxidase enzyme leads to the formation of theaflavins (TF) and thearubigins (TR)^7^. Theaflavins which are responsible for the taste, brightness and contribute to the colour of black teas. Thearubigins are responsible for thickness and colour of both the liquors and infusion^8^. The other plain tea quality parameters or attributes, include black tea brightness, astringency, colour, briskiness and aroma^9^. Indeed, theaflavins have become a critical parameter in estimating the quality and the price of black teas^10^. Caffeine is important in cream formation and the briskness of tea and therefore, has been used in predicting black tea quality^11,12^. Polyphenols and caffeine biosynthesis are dependent on structural and regulatory genes; structural genes encode enzymes catalyzing the biosynthesis, while regulatory genes control the expression of the genes.

Drought is a major factor which significantly influences the growth and production of tea^13,14^. Hence, breeding drought-tolerant tea cultivars that have high recoverability potential is an important target for tea breeders. Although, the morphological and physiological mechanisms of drought tolerance in tea plant has been reported, only few reports are available on the changes of genes related to catechin biosynthesis in response to environmental stresses^15,16^. This is because drought tolerance is a complex multigenic trait, and various regulatory and functional genes are involved. In tea plant^17^, reported that polyphenols can be used as a potential indicator for drought tolerance. In another study, results suggested epicatechin and epigallocatechin as potential indicators in predicting drought tolerance in tea plant^18^. Recently, transcriptomic analysis of drought susceptible and drought-tolerant tea plants during drought stress has been reported^15,19,20^. The molecular responses of plants to drought stress include perception, signal transduction, gene expression, and ultimately metabolic changes that lead to stress tolerance. The induced drought stress lead to gene expression which participate in the generation of hormones like abscisic acid (ABA), salicylic acid, and ethylene. For example, ABA has been shown to play crucial roles in regulating the drought response in many plants, and its metabolic pathway involves multiple steps and genes^21^. The level of ABA in plants increases rapidly during drought stress, leading to stomatal closure and induction of stress genes to cope with the stress^22^. Drought stress also results in accumulation of proline, mannitol, sorbitol, formation of radical scavenging compounds (ascorbate, glutathione, tocopherol), and synthesis of protein^23,24^. Proline acts as an osmoprotectant and its accumulation is induced to tolerance of tea to water deficit stress, as this helps in maintaining water relations, prevents membrane distortion, and also acts as a hydroxyl radical scavenger^24^. The increase in generation of reactive oxygen species (ROS) such as superoxide radicals (O2), hydrogen peroxide (H2O2), hydroxyl radical (.OH), alkoxyl radical (.RO), and singlet oxygen (1O2) within the cell, leads to lipid peroxidation, protein degradation, and enzyme inactivation and affects nucleic acid leading to cell death^23,24^. The objective of the current study was to annotate and assign biosynthetic pathway functions to putative QTLs linked to caffeine content, catechins fractions, theaflavins fractions, tea taster scores and %RWC.

## Materials and Methods

### Preparation of FASTA files

The sequences of DArTseq- based markers were derived from the tag sequence associated with each DArTseq marker as explained in the previous work^9^ and were generated by Diversity Arrays Technology Pty. Ltd. (Canberra, Australia). Marker sequences were arranged in FASTA format by starting with a single-line description of the sequence, followed by sequence data. The single-line description was distinguished from the sequence data by placing a symbol “ > ” in front of the description.

### BLAST search

A total of 1,421 DArTseq markers on LG1 to LG15 were subjected to a BLAST search. The marker tag sequences (FASTA format) derived from the DArTseq map were searched using the non-reductant BLASTN program against the assembled draft tea genome (*Camellia sinensis* var *assamica*)^1^. The best hit was then selected based on E-value and % identity. The E-value served as a measure of the number of hits one can “expect” to see by chance when searching a database with small E-value indicating homology. The % identity showed the percentage of identical residues between query sequences and hit sequences from a database, with longer stretches of homology more likely to indicate a genuine match.

### Functional annotation and pathway assignment

Functional annotation of all 1,421 DArTseq markers was performed on BLASTX search against the non-redundant GenBank protein sequence database with a threshold E-value of 10-6^25,26^. Functional annotation and mapping of gene ontology (GO) terms was done with the Blast2GO program (Blast2GO v3.2)^27^. Each BLAST hit associated with the GO terms were retrieved and annotated using the following annotation score parameters; E-Value Hit Filter (default = 1.0E-6), GO-Weight (default = 5), Hsp-Hit Coverage Cut Off (default = 0), Annotation Cut-Off (90). The contig sequences were also queried for conserved domains/motifs using InterProScan, which is an on-line sequence search plug-in within the Blast2GO program. The Kyoto Encyclopedia of Genes and Genomes (KEGG) mapping was used to determine metabolic pathways. The sequences with corresponding evidence code (EC) numbers obtained from Blast2GO were mapped to the KEGG metabolic pathway database.

## Results

### Functional annotation and pathway assignment

The identification of candidate genes for tea was conducted in all the 15 LGs derived from the DArTseq maps. Sequences of each marker on all the LGs were subjected to a BLAST search against tea genome BLAST database in an effort to compare the homology and identify the location of the gene of interest within the tea genome. The results of BLAST searches for markers derived from the DArTseq map are presented in Table 1. Of 1,421 DArTseq markers derived from DArTseq map, 53 markers were identified to be link to black tea quality and %RWC. A total of seven markers were dominant in TRFK 303/577 parent while 9 markers were dominant in GW Ejulu parent, respectively. All the 53 markers with unique best hits were identified in the tea genome. The 23 markers showed locations across ten chromosomes in tea, which were LG1 (1), LG3 (1), LG4 (8), LG6 (1), LG9 (1), LG10 (2), LG12 (2), LG13 (4) LG14 (2) and LG15 (1). A total of 14 markers were dominant, 8 were dominant in GW Ejulu and six were dominant in TRFK 303/577, respectively. The dominant markers in GW Ejulu were for caffeine, catechin, ECG, EGC and EGCG. On the other hand, the dominant markers in TRFK 303/577 were for caffeine, ECG, EGC, TF1 and %RWC. All the 13 dominant markers were identified in the tea genome for the two parents used in this study as reciprocal crosses in the two tea populations (Table 1)

**Table 1:** Functional annotation of putative candidate genes in 15 linkage groups of *C*. *sinensi*s on reference tea genome.

Nr	QTL	Parent	DArTseq Marker	LG	Position (cM)	LOD	PEV	E-value	Annotated protein	Function
1	qECG^a^	TRFK 303/577, GW Ejulu	5128890	1	96.4	6.8	11.7	2.0E-25	[‘Actin’]	Abiotic stress
2	qEC^a^	TRFK 303/577, GW Ejulu	5072338	2	2.2	4.1	5.6	2.0E-25	[‘Peptidase family M3’]	Abiotic stress
3	qEGC^a^	GW Ejulu	5124128	2	7.7	3.2	6.8	2.0E-18	[‘Kinesin motor domain’]	Transport protein
4	qCaffeine^b^	TRFK 303/577, GW Ejulu	5064585	2	50.9	3.4	5.8	6.0E-25	[‘Peptidase C65 Otubain’]	Modification of cellular proteins
5	qECG^a^	TRFK 303/577, GW Ejulu	5097659	4	17.1	4.6	7.8	1.0E-07	[‘Rpp14/Pop5 family’]	—
6	qECG^a^	TRFK 303/577	5087113	4	17.7	4.7	22.9	6.0E-22	[‘impB/mucB/samB family’]	UV protection through DNA repair
7	qEC^a^	GW Ejulu	5134490	4	26.4	12.6	43.7	3.0E-08	[‘Aminotransferase class I and II’]	Phenylalanine, tyrosine and tryptophan biosynthesis
8	qEGCG^a^	GW Ejulu	5134853	4	37.6	14.9	45.1	2.0E-06	[‘Diacylglycerol kinase catalytic domain’]	Abiotic stress
9	qTF1^a^	TRFK 303/577, GW Ejulu	5106352	4	26.0	14.7	45.6	2.0E-06	[‘Thiolase, C-terminal domain’]	Benzoic acid biosynthesis
10	qEC^a^	GW Ejulu	5123475	4	27.2	14	51.5	1.0E-10	CSA016461	—
11	qEGC^a^	GW Ejulu	5123475	4	27.2	3.7	51.5	1.0E-10	CSA016461	—
12	qEC^a^	TRFK 303/577	5119221	4	32.7	3.1	53.8	1.0E-19	[‘Histone acetyltransferase subunit NuA4’]	Drought response
13	qEGCG^a^	TRFK 303/577	5119221	4	20.6	3.3	53.8	1.0E-19	[‘Histone acetyltransferase subunit NuA4’]	Drought response
14	qEGC^a^	TRFK 303/577, GW Ejulu	5136058	4	27.6	46.1	54.1	1.0E-07	[‘Autophagy-related protein 11’]	Abiotic stress
15	qTF4^a^	TRFK 303/577, GW Ejulu	5136058	4	27.6	14.8	54.1	1.0E-07	CSA024230	—
16	qCaffeine^b^	TRFK 303/577, GW Ejulu	5114692	4	68.6	3.8	6.1	4.0E-23	[‘BT1 family’]	Transport protein
17	qCAT^b^	TRFK 303/577	5119221	4	32.7	3.1	2.3	1.0E-19	[‘Histone acetyltransferase subunit NuA4’]	Drought response
18	qECG^a^	TRFK 303/577	5136985	6	26.4	5.1	5.3	6.0E-28	[‘KOW motif’]	—
19	qTF1^a^	TRFK 303/577	5136045	6	69.6	4.5	6.9	6.0E-19	[‘Catalase’]	Abiotic stress
20	qECG^b^	TRFK 303/577, GW Ejulu	5108503	6	56.5	4.8	8.2	2.0E-27	[‘DnaJ domain’]	Drought response
21	qECG^b^	GW Ejulu	5098382	6	56.9	4	8.7	6.0E-19	[‘Asparagine synthase, Glutamine amidotransferase domain’]	Nitrogen mobilization
22	qRWC^b^	TRFK 303/577	5082606	6	66.2	3.3	5.7	9.0E-21	[‘Alpha adaptin AP2’]	Abiotic stress
23	qCaffeine^b^	GW Ejulu	5064391	7	48.1	3.7	6	2.0E-27	[‘Lipase (class 3)’]	Lipid degradation, esterification and transesterification processes
24	qCaffeine^b^	TRFK 303/577, GW Ejulu	5134558	8	18.8	3.9	7.5	9.0E-24	[‘Nitronate monooxygenase’]	Catabolic or anabolic pathways
25	qRWC^b^	TRFK 303/577, GW Ejulu	5130531	9	6.7	4	7	7.0E-06	[‘MatE’]	Drought response, Sequestration of proanthocyanidins
26	qTF2^a^	TRFK 303/577, GW Ejulu	5128967	10	28.7	3.5	7	2.0E-25	[‘Acyl-CoA oxidase’]	Lipid catabolism and plant hormone biosynthesis
27	qECG^a^	GW Ejulu	5072021	10	25.5	4.3	7.5	8.0E-09	[‘ATPase family associated with various cellular activities (AAA)’]	Heat stress response
28	qECG^b^	GW Ejulu	5136108	10	20.6	4.8	5.7	1.0E-10	[‘Protein kinase domain’]	Abiotic stress
29	qECG^b^	TRFK 303/577, GW Ejulu	5124207	10	20.4	3.1	5.3	8.0E-12	[‘Acyltransferase’]	Phenylpropanoid and Shikimate pathway
30	qEGCG^a^	TRFK 303/577	5088456	12	47.9	3.9	9.8	4.0E-23	[‘Protein kinase domain’]	Abiotic stress
31	qCAT^a^	GW Ejulu	5136077	12	42.8	6.6	11.2	5.0E-13	CSA026168	—
32	qEGC^a^	GW Ejulu	5136077	12	42.8	5.6	11.2	5.0E-13	CSA026168	—
33	qCAT^a^	TRFK 303/577, GW Ejulu	5123751	12	43.0	6.1	11.5	2.0E-18	[‘Bromodomain’]	Scaffolding proteins
34	qEGC^a^	TRFK 303/577, GW Ejulu	5123751	12	43.0	14.9	11.5	2.0E-18	[‘Bromodomain’]	Scaffolding proteins
35	qCaffeine^b^	TRFK 303/577, GW Ejulu	5088162	13	29.7	4.7	5.4	6.0E-28	[‘PA domain’]	—
36	qCAT^b^	TRFK 303/577	5103784	13	50.6	3.8	6.1	8.0E-12	CSA003424	—
37	qCAT^b^	TRFK 303/577	5122899	13	50.5	3.8	6	6.0E-19	CSA033214	—
38	qCAT^b^	TRFK 303/577, GW Ejulu	5133009	13	48.6	3.6	5.8	1.0E-16	[‘Adaptor complexes medium subunit family’]	—
39	qCAT^b^	TRFK 303/577	5122899	13	50.5	3.8	6	6.0E-22	[‘Protein kinase domain’]	Abiotic stress
40	qCAT^b^	TRFK 303/577	5122899	13	50.5	3.8	6	2.0E-21	[‘14-3-3 protein’]	Abiotic stress
41	qCAT^b^	TRFK 303/577	5122899	13	50.5	3.8	6	2.0E-21	[‘NB-ARC domain’]	Disease resistance
42	qCAT^b^	TRFK 303/577	5122899	13	50.5	3.8	6	2.0E-21	[‘Pectinesterase’]	Drought response
43	qCAT^b^	TRFK 303/577	5122899	13	50.5	3.8	6	1.0E-16	[‘2OG-Fe(II) oxygenase superfamily’]	Abiotic stress
44	qCAT^b^	TRFK 303/577, GW Ejulu	5111268	13	50.6	4.1	7.2	6.0E-28	[‘WD domain’]	Transport protein
45	qCAT^b^	TRFK 303/577, GW Ejulu	5123751	13	58.1	4.1	6.5	4.0E-23	[‘Transmembrane amino acid transporter protein’]	Abiotic stress
46	qECG^b^	TRFK 303/577, GW Ejulu	5088162	13	50.6	3.7	5.4	6.0E-28	[‘PA domain’]	—
47	qEGC^b^	TRFK 303/577, GW Ejulu	5123751	13	50.6	3.7	6.5	4.0E-23	[‘Transmembrane amino acid transporter protein’]	Abiotic stress
48	qEGC^a^	GW Ejulu	5116677	14	63.1	4.1	5.2	6.0E-28	CSA002263	—
49	qEGC^a^	GW Ejulu	5116677	14	63.1	4.1	5.2	3.0E-11	[‘Armadillo/beta-catenin-like repeat’]	Heat stress response
50	qBRT^a^	TRFK 303/577	5122986	14	65.4	3.7	7.5	7.0E-06	[‘Glycosyl hydrolase family 9’]	Phenylpropanoid pathway
51	qCAT^b^	—	5132370	14	60.7	4.1	2.5	1.0E-10	[‘Glutaminyl-tRNA synthetase’]	Chlorophyll biosynthesis
52	qECG^b^	GW Ejulu	5111164	15	75.1	4.2	7.2	1.0E-07	[‘Isocitrate/isopropylmalate dehydrogenase’]	Abiotic stress
53	qEGCG^b^	TRFK 303/577, GW Ejulu	5114089	15	32.1	3.8	6.8	8.0E-12	[‘Cytochrome P450’]	Biotic and abiotic stresses

^a^Putative QTL identified based on Interval Mapping with LOD >3.0.

^b^Putative QTL identified based on Multiple QTL Model Mapping with LOD >3.0.

QTL- Quantitative trait loci; LG- Linkage group; LOD- Logarithm of odd ration; cM- Centimorgan, CAFF Caffeine, CAT Catechin, EC epicatechin, ECG epicatechin gallate, EGC epigallocatechin, EGCG epigallocatechin gallate, TF1 theaflavin, TF4- Theaflavin-3,3′-digallate, BRT brightness, %RWC percent relative water content.

### Annotation and mapping of gene ontology

**Figure 1:**
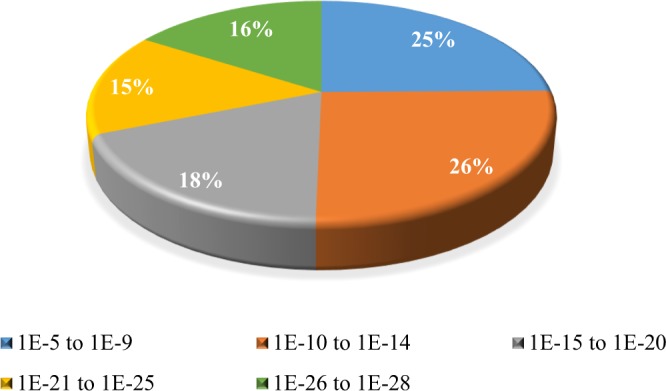
E-value distribution of the best matches for unigenes (E-value ≤ 1.0e-5).

**Figure 2:**
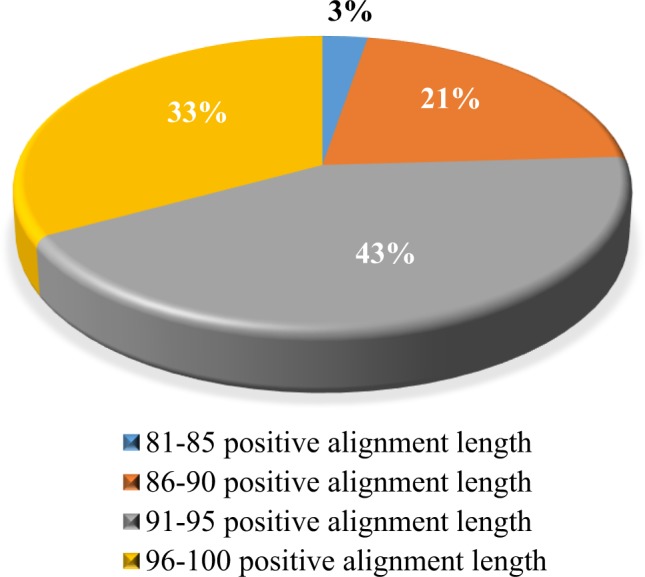
Similarity distribution of the hit BLAST matches for unigenes.

All unigenes in this study were assigned GO terms based on BLASTX searches against the non-redundant (NR) databases (Fig. 1 and 2). Our results showed that 86–100% similarity distribution of the top hits in the non-redundant (NR) database (Fig. 1). The E-value distribution of the mapped sequences with high homologies (smaller than 1e-6) was high as compared to homologous sequences that were greater than 1e-5 (Fig. 2). The E-value distribution of the best hits in the NR database showed that 26% annotated sequences had high identity with their best hits (smaller than between 1e-28 to 1-e26), whereas 15% ranged from 1e-21 to 1e-25 and another 18% ranged from 1e-15 to 1e-20 (Fig. 1). The similarity distribution revealed that 43% of the query sequences had greater than 91–95% similarity, whereas 21% of the query sequences varied in similarity from 86–90%, and the other 3% varied from 81–85% (Fig. 2).

**Figure 3:**
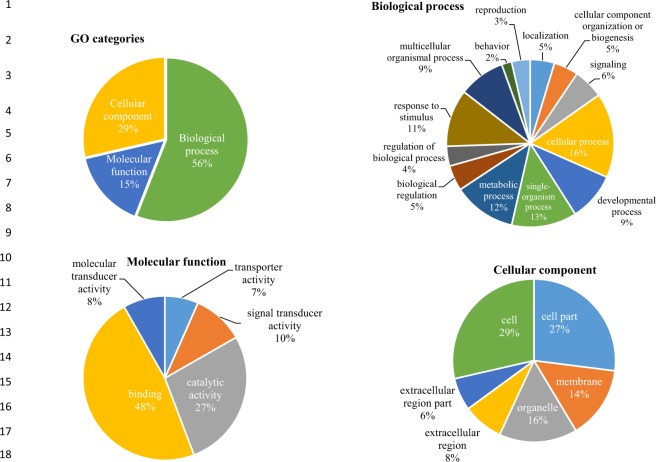
Function classifications of GO terms of *C*. *sinensis* transcripts based on high-score BLASTX matches in NR plant proteins database, a total of 5,592 unigenes were classified into three main GO categories which are biological process (BP), molecular function (MF) and cellular component (CC), respectively.

A total of 5,592 unigenes were assigned GO terms, of these, biological processes (56%) comprised the largest category, followed by cellular component (29%) and molecular functions (15%), respectively (Fig. 3). In biological process (BP) category, the majority of unigenes were involved in regulation of biological process (35%), cellular process (16%), metabolic process (12%), response to stimulus (11%), developmental process (9%) and localization and signaling (6%), respectively (Fig. 3). The cellular and metabolic processes were the most highly represented groups in the biological process, which suggests that extensive metabolic activities taking place in the fresh tea flush. The best-represented groups of molecular function (MF) were binding activity (48%), catalytic activity (27%), signal transducer activity (10%), molecular transducer activity (8%) and transporter activity (7%), respectively (Fig. 3). Within the molecular function category, binding and catalytic activities were the most abundant groups. The cellular component (CC) included the cell (21%), cell part (21%), organelle (16%), membrane (11%) organelle part (7%), membrane part (7%), and macromolecular complex (6%), respectively (Fig. 3). The transcripts that corresponded to cell, cell parts, and cell organelles were the most abundant groups within the cellular component category. Among the top 20 functional sub-groups in biological processes, multicellular organism development, response to stress and response to endogenous stimulus represented the largest group (Supplementary Fig. 1). The top 20 functional sub-groups for molecular functions, protein binding, receptor binding and signal transducer activity represented the largest group (Supplementary Fig. 2), while plasma membrane, nucleus and cytoplasm were the largest group that represented the top 20 sub-groups of cellular component (Supplementary Fig. 3).

### KEGG pathway mapping: Flavonoid pathway, caffeine pathway and abiotic stress

**Figure 4:**
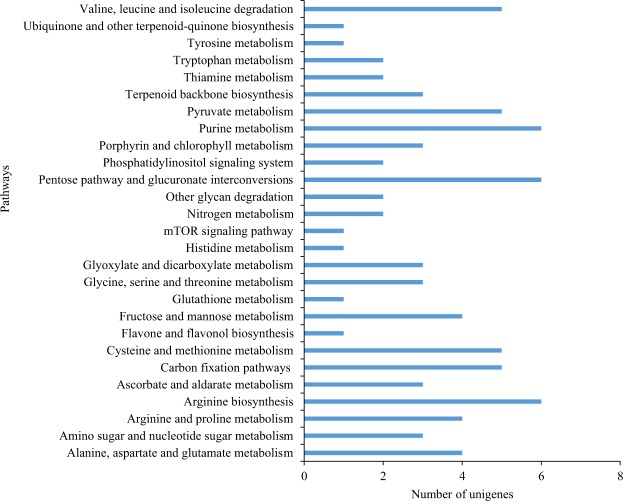
KEGG ontology (KO) analysis of differently expressed genes (DEGs) related to secondary metabolism in *C*. *sinensis*.

Pathway-based analysis for the tea flush transcriptome is helpful to further understand the biological functions of genes involved in the tea flavonoid biosynthesis pathway, caffeine pathway and stimulus to response to abiotic stress (drought stress). A total of 53 unigenes linked to putative QTL were assigned two different KEGG database pathways (Supplementary Table 1) while 84 unigenes in 15 linkage groups were assigned to 25 different KEGG database pathways based on categories of secondary metabolite biosynthesis (Supplementary Table 2, Fig. 4). The pathways with most representations were metabolic pathways and biosynthesis of secondary metabolites, which was consistent with the GO categories of the biological process (Fig. 3 and Supplementary Fig. 1). Of these, 52 unigenes were categorized into the metabolism groups, with most of them involved in purine metabolism (11%), cysteine and methionine metabolism (9.6%), pyruvate metabolism (9.6%), alanine, aspartate and glutamate metabolism (7.6%), arginine and proline metabolism (7.6%) and fructose and mannose metabolism (7.6%) and other sub-categories, respectively. The most abundant unigenes were for carbohydrate and amino acids biosynthesis, which are mostly involved in plant hormone signal transduction pathways in response to abiotic stress and biosynthesis of other secondary metabolites such as phenylpropanoid biosynthesis, flavonoid biosynthesis and alkaloid biosynthesis. The unigenes associated with flavone and flavonol biosynthesis, a key regulatory pathways in the metabolism of the main chemical components in *C*. *sinensis* leaves were present (Fig. 4). Unigenes associated with purine metabolism, also a key regulatory pathway which is involved in metabolism of adenine and guanosine nucleotides were present.

**Figure 5:**
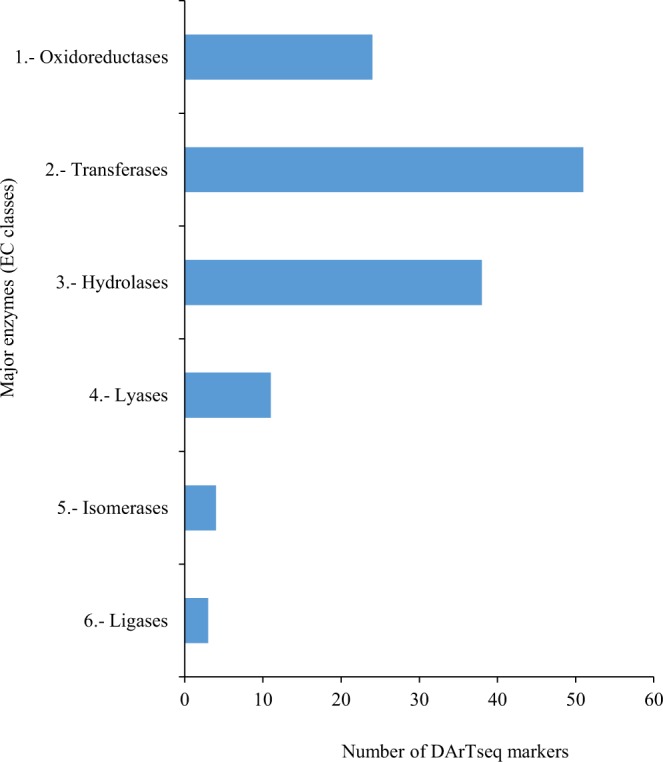
KEGG ontology (KO) distribution of major enzymes code related to secondary metabolism in *C*. *sinensis*.

Also, major enzymes that are involved in different metabolic pathways were further grouped into the six different categories; oxidoreductases, transferases, hydrolases, lyases, isomerases and ligases. The distribution of the three abundant enzymes was also consistent with the GO categories of molecular function (Fig. 3 and Supplementary Fig. 2). The three major enzymes distribution included; transferase enzymes (38.9%), which are a class of enzymes that catalyze the transfer of specific functional groups (a methyl, or glycosyl group, or galloyl group) from a donor to acceptor molecules, hydrolase enzymes (29%), which catalyze the hydrolysis of compounds and oxidoreductase enzymes (18.3%), which catalyze the oxidation-reduction reactions (Fig. 5 and Supplementary Fig. 3). The three other enzymes that were less abundant in distribution included; lyase enzymes (8.4%), which cleave carbon-carbon, carbon-oxygen, phosphorous-oxygen and carbon-nitrogen bonds without hydrolysis or oxidation reactions, isomerase enzymes (3.1%), convert a molecule from one isomer to another while ligase enzymes (2.3%), catalyze the formation of a new chemical bond by joining of two large molecules (Fig. 5).

### Functional annotation of putative candidate genes 15 linkage groups of *C*. *sinensis*

The 28% of the mapped sequences (405 out of 1,471) were annotated. Functional annotations of detected QTL led to the identification of functions of 37 putative candidate genes in parental clones that could be involved in the expression of the targeted traits (Table 1). The putative candidate genes involved in secondary metabolism (phenylpropanoid biosynthesis, flavonoid biosynthesis, alkaloid biosynthesis and volatile compounds biosynthesis) were located in five linkage groups LG04, LG09, LG10 and LG13. The putative candidate genes identified were involved in phenylpropanoid and flavonoid biosynthesis pathways included a group or class of enzymes namely; 2-oxoglutarate/Fe(II)-dependent dioxygenases superfamily (2-ODDs), aminotransferase class I and II, acyltransferase, glycosyl hydrolase family 9. This was in agreement with KEGG pathway mapping where enzymes mapped clustered into six major groups according to different metabolic pathways they are involved with transferases, hydrolases and oxidoreductases as three abundant enzymes. The multidrug and toxic compound extrusion (MATE) transporter was also putatively identified in LG09. BT1 family protein located in LG04 was putatively annotated for qCAFF. The putative QTL and their corresponding putative candidate genes associated with phenylpropanoid biosynthesis, and flavonoid biosynthesis were identified in both parental clones.

Proteins related with oxidative stress and ATPase family proteins or proteins related to abiotic stress were found in the confidence intervals of QTL for drought tolerance traits. Among the 53 examined putative QTL in parental clones (Table 1), 18 putative QTL were related to abiotic stress (ABA signaling, amino acids peptide degradation) and were mostly found on LG04 and LG13. However, the putative QTL related to abiotic stress, including percent RWC was also found on LG01, LG06, LG10, LG12, LG14 and LG09, respectively. The putative candidate genes identified included, pectinesterase, 14-3-3 protein, 2OG-Fe (II) oxygenase superfamily, protein kinase domain, transmembrane amino acid transporter protein, peptidase family M3, histone acetyltransferase subunit NuA4, DnaJ domain and MatE. ATPase family proteins associated with various cellular activities (AAA), and Armadillo/beta-catenin-like repeat protein were found to be associated with the heat stress response. The putative QTL in LG13 that was associated with disease stress response was NB-ARC domain protein. Putative candidate genes related with ABA signaling pathway were found on QTL for qCAT, qEC, qEGCG and qRWC (Table 1), respectively. The putative candidate gene related to degradation of peptides by targeting the amino acids residues that are formed during abiotic stress was found on QTL for qEC. The parental clone TRFK 303/577 which is known to be high yielding and drought-tolerant cultivar, exhibited most putative candidate genes related to drought stress response as compared to parental clone GW- Ejulu.

## Discussion

Flavonoid biosynthesis pathway in tea begins with the amino acid phenylalanine, which undergoes deamination to form trans-cinnamic acid^28,29^. The oxidation process of trans-cinnamic acid yields p-coumaric acid, which undergoes transformation to form p-coumaroyl-CoA. The committed step in flavonoid biosynthesis is the subsequent condensation of p-coumaroyl-CoA and malonyl-CoA catalyzed by chalcone synthase enzyme to form chalcone. The subsequent isomerization of chalcone forms (2S)-flavonones. The identification of putative QTL, qCAT, for 2-ODDs superfamily protein in this study corroborates with other previous reports on the role of 2-ODDs in flavonoid biosynthesis. The 2-ODDs which are large superfamily of non-heme proteins, which facilitate numerous oxidative reactions, including hydroxylation, epimerization, C-C bond cleavage, ring formation and fragmentation. These 2-ODDs superfamily catalyze key oxidative reactions that facilitate the formation of different flavonoid subclasses, for example, the oxidation of (2S)-flavonones. The (2S)-flavonones are converted to dihydroflavonols through hydroxylation process which is then catalyzed by another 2-ODD, flavonone 3β-hydroxylase (F3H). The dihydroflavonols formed is a substrate for flavonol synthase (FNS I), which competes with dihydroflavonol 4-reductase (DR4) to form flavonols, anthocyanidins and procyanidins.

Glycosylation is an important process for the diverse functions of polyphenolic compounds in plants and is known to be able to increase the solubility and stability of hydrophobic flavonoids^30^. The glycosylation process is regulated by glycoside hydrolases, which are involved in hydrolysis and/or rearrangement of glycosidic bonds. We hypothesize the putative QTL, qBRT linked to glycosyl hydrolase family 9, a family of glycoside hydrolases in the current study to play a role in the synthesis and sequestration of some phenylpropanoids^31^. In tea, the galloylated catechins formed through the glucosylation activity of UDP-glucosyltransferase, have been found to confer astringent and bitter tastes, while flavonol 3-O-glycosides have been found to induce velvety, mouth-drying, and mouth-coating sensations^6^. In legume plant, bur clover (*Medicago truncatula*), epicatechin 3′-O-glucoside, has also been shown to be involved in the biosynthesis of proanthocyanidin in the seed coat^32,33^. In addition, the glycosylated polyphenols function also as acyl donors in biochemical reactions, for example, β-glucogallin, a glucose ester of gallic acid, functions as an acyl transfer donor in the biosynthesis of both galloylated tannins^34^ and galloylated catechins^35^.

Acyltransferases which participates in many secondary metabolism pathways was putatively identified in this current study (qECG) in LG10. Acyltransferases are involved in the biosynthesis of anthocyanidin and other flavonoid groups by transferring acyl groups to the sugar moiety of anthocyanins using acyl-CoA as the donor^36^. The process of modification of anthocyanin and other flavonoid groups with acyl groups is catalyzed by acyltransferases and is important in maintaining their stability. In LG04, qCAFF, was putatively annotated as BT1 family protein which is has been identified to be involved in export of adenine nucleotides, which are exclusively synthesized in plants plastids, and are precursors for caffeine biosynthesis^37^. Furthermore, nucleotides, adenine and guanosine are precursors in caffeine biosynthesis in tea plants^38^.

The 14-3-3 proteins which bind to other proteins to induce target-site specific alteration and conformation of target proteins are important plant signal transduction pathways. Previous reports implicate plant 14-3-3s in key physiological processes, in particular, abiotic and biotic stress responses, metabolism (especially primary carbon and nitrogen metabolism), as well as various aspects of plant growth and development. In the current study, putative QTL, qCAT in LG13 was putatively annotated 14-3-3 protein, which is abiotic or biotic stress protein in tea. This, therefore, is in agreement with previous study by^18^ where individual catechins were demonstrated as potential indicators in predicting drought tolerance in tea plant. Meanwhile, a number of recent studies in *Arabidopsis* have linked 14-3-3s to ABA signaling. Some of the main functions ascribed to ABA include response to abiotic/biotic stress.

Drought stress which usually affects the plant photosynthetic process also interferes with plant nutrient availability leading to ion intoxication. The reversible protein phosphorylation by protein kinases at the early and later stage of the signaling pathways is one of the mechanisms that plants use in response to abiotic stress. In addition, to maintain the osmotic homeostasis and tolerance due to drought stress, plants also do regulate gene expression of some specific genes of protein kinases. The presence of a putative QTL, qCAT, which was annotated for protein kinase domain in this study supports the argument of the previous studies on the role of tea catechins as potential indicators of drought tolerance in tea plants. A previous study by^39^ indicated that catechins were higher in the drought-tolerant cultivars and therefore, corroborated with the presence of qCAT, annotated as protein kinase domain in drought-tolerant parental clone TRFK 303/577.

Transmembrane amino acid transporter protein functions as gateways to permit the transport of amino acids across the biological membrane. Amino acids are not only critical for the synthesis of proteins, but other many essential compounds, which act as signal molecules. For example, in *Arabidopsis*, water and salt stress induce a strong expression of proline transporter 1 (ProT1) and proline transporter two while the expression of amino acid permease 4 (AAP4) and amino acid permease 6 (AAP6) is repressed^40,41^. In tea, proline has been shown to act as an osmo-protectant during drought stress^23,24^ and the presence of a putative candidate gene (transmembrane amino acid protein) in this current study, could hypothesize the functions of transmembrane amino acid transporter for proline presence during drought stress. Furthermore, the presence of transmembrane amino acid transporter proteins could be their involvement of transport of aromatic amino acids phenylalanine, tyrosine and tryptophan, which serve as precursors for a wide range of secondary metabolites (polyphenols) in tea. This is because the two identified putative QTL, qCAT and qEGC in LG13 in both parental clones (TRFK 303/577 and GW Ejulu) are products of shikimate and phenylalanine pathways.

The MATE proteins are a family of secondary active transporters, which utilize electrochemical gradient of membrane maintained by ATPases for their transport activity. MATE transporters are involved in transport of secondary metabolites into vacuoles, which are the major storage site of most of the conjugated flavonoids, which comprise of flavonols, anthocyanins and flavone glycoside^42^. In the current study, putative QTL, qRWC in LG09 was annotated for MATE proteins, which are involved in the sequestration of flavonoids in vacuoles in response to drought stress^43^. Secondary metabolites in plants, especially flavonoids play an important role in response to abiotic stress such as drought stress. A member of MATE gene family (TT12) has also been elucidated for sequestration of proanthocyanidins in seed vacuoles, which leads to pigmentation of the seed coat^44^. In yeast, it was revealed that TT12 proteins can specifically transport glycosidic form like epicatechin 3′-O-glucoside and cyanidin 3-O glucoside^45^. The MATE proteins from a legume, bur clover (*Medicago truncatula*) and grapevines (*Vitis*) have been characterized for transport of flavonoids through the sequestration of epicatechin 3-O-glucoside and cyanidin 3-O glucoside (proathocyanidins) into the vacuole for storage^33^. In another study, a part from the role of vacuolar sequestration flavonoids, MATE proteins have been recognized in transport of alkaloids into vacuoles in tobacco. Furthermore, MATE proteins have facilitated and modulated abscisic acid efflux and sensitivity in drought-tolerant *Arabidopsis*^46^. Since a putative QTL for %RWC annotated as MATE in this current study, it therefore, corroborates with previous reports^47^ which relied on the percent %RWC to classify tea cultivars as either drought tolerant or drought susceptible based on tea leaves metabolites.

In this current study, putative QTL, qECG in LG06 was putatively annotated DnaJ protein. DnaJ proteins are essential components that contribute to cellular protein homeostasis and complex stabilization of proteins (protein folding, degradation and refolding) under stress conditions in plants^48^. They function as molecular chaperones either alone or in association with heat-shock protein 70 (Hsp70). In a previous study on the transcriptomic analysis of the effect of drought-induced-stress on tea leaf quality, revealed that the levels of ECG and EGCG increased during drought stress^49^. This was also reported by^39^ on the effects of soil moisture stress alterations on tea biomolecules in relation to tea quality. Furthermore, in transgenic tobacco, the overexpression of tomato chloroplast-targeted DnaJ enhanced drought tolerance^50^ and maintained photosystem II under chilling stress^51^. Our findings, therefore, suggest that ECG can be used as a potential marker of drought tolerance or cold stress in tea cultivars.

The putative QTL, qEGCG, in LG04 was annotated diacylglycerol kinase catalytic domain protein which we hypothesize as a marker for drought and cold tolerance in tea cultivars. Abiotic stress, including salinity, drought, and osmotic adjustments triggers the production of phosphatidic acid^52^. Phosphatidic acid is formed by phosphorylation of diacylglycerol by diacylglycerol kinase. Thus, phosphatidic acid rather than diacylglycerol has typically been implicated as a major plant secondary messenger. However, the presence of diacylglycerol is necessary for certain developmental processes and the response to particular environmental stimuli. For example, salinity stress in *A*. *thaliana* increased the activity of non-specific phospholipase C, which promoted the production of diacylglycerol. In addition, cold stress has recently been found to stimulate the formation of phosphatidic acid in suspension-cultured *Arabidopsis* cell^53^.

Histone acetyltransferases play a critical role in histone acetylation of plant chromatin, which is important in epigenetic control of gene expression. The transfer of acetyl groups to core histone tails by histone acetyltransferases, promote transcription of target genes involved in drought response and absisic acid signaling in several plants, including *Arabidopsis*^54^, rice^55^, and maize^56^. Furthermore, previous studies reported up-regulation of drought stress responsive genes and found to be correlated with changes in histone modification^57,58^. The putative QTL, qCAT, qEC and qEGCG in this current study were annotated histone acetyltransferase subunit NuA4 protein in drought-tolerant parental clone TRFK 303/577. Therefore, catechins could act as potential indicators or markers of drought tolerance in tea, which corroborates with previous reports^18,39^ which investigated the biochemical changes in tea constituents as influenced by varying levels of water stress.

Aminotransferases, also known as transaminases, catalyze amino group transfers from amino donor to amino acceptor compounds. Aminotransferases play a major role in a variety of metabolic pathways, including, amino acid biosynthesis, secondary metabolites biosynthesis and photorespiration. Prephenate aminotransferase, a class of aminotransferase enzyme, catalyze the final step in the synthesis of phenylalanine, which is the central product of shikimic acid pathway and serves as a precursor for the synthesis of flavonoids^59^. The identification of putative QTL, qEC in the current study annotated as aminotransferase I and II is hypothesized as a marker linked to flavonoids biosynthesis in tea.

## Conclusion

This current study is the first attempt to obtain more information on genomic and functional annotation of proteins/ enzymes involved in black tea quality and drought tolerance traits in tea plant based on DArTseq markers. The DArTseq markers used, and putative QTL identified in this current study will shed more light on the proteins/ enzymes associated with the biosynthesis of caffeine, individual catechins in green tea which are converted to individual theaflavins in black tea processing. In addition, the DArTseq markers will provide useful information, in particular for studies of the genetic determinants of black tea quality traits and drought tolerance in tea. Also, the DArTseq markers used in the current study can be useful markers for construction of linkage maps or may be used to discover new functions of genes. We successfully constructed a genetic linkage map using SNPs, but it was impossible to anchor it to other previously constructed linkage maps of tea since no anchoring markers were available. Furthermore, it was also difficult to deduce the relative genetic positions of the SNPs on the previous different linkage maps. Indeed, previous studies used RAPD, AFLP and SSRs markers to construct tea genetic linkage maps, while in our study, we used DArTseq markers. However, the information obtained through the annotation of putative gene functions and the alignment of DArTseq sequences relative to recently published reference tea genome, has set a milestone for a future and further researches on marker-assisted selection and breeding of tea plants. In addition, this work may help breeders in selection of parents with desirable DArTseq markers for development of new tea cultivars with desirable traits.

## Supplementary information

Supplementary Figures

## Data Availability

The DArT sequences have been submitted to NCBI (http://www.ncbi.nlm.nih.gov/). BioProject PRJNA398959.
