# Supplementary material for: Functional annotation of putative QTL associated with black tea quality and drought tolerance traits
Source: Sci Rep. 2019 Feb 6;9:1465. doi: 10.1038/s41598-018-37688-z (PMC6365519; doi:10.1038/s41598-018-37688-z)
Supplement: Supplementary file 1 — Supplementary Figures [file 41598_2018_37688_MOESM1_ESM.pdf]

ELECTRONIC SUPPLEMENTARY MATERIALS

The following information accompanies the article:

Functional annotation of putative QTL associated with black tea quality and drought tolerance traits

Robert. K. Koech[1], [2], Pelly M. Malebe[1], Christopher Nyarukowa[1], Richard Mose[3], Samson M. Kamunya[2], Fourie Joubert[1] and Zeno Apostolides[1]*
[1]Department of Biochemistry, Genetics and Microbiology, University of Pretoria, Pretoria 0002, South Africa
[2]Kenya Agriculture and Livestock Research Organization, Tea Research Institute, P.O. Box 820, Kericho 20200, Kenya
[3]James Finlay (Kenya) Limited, P.O. Box 223, Kericho 20200, Kenya.
*Corresponding author: za@up.ac.za

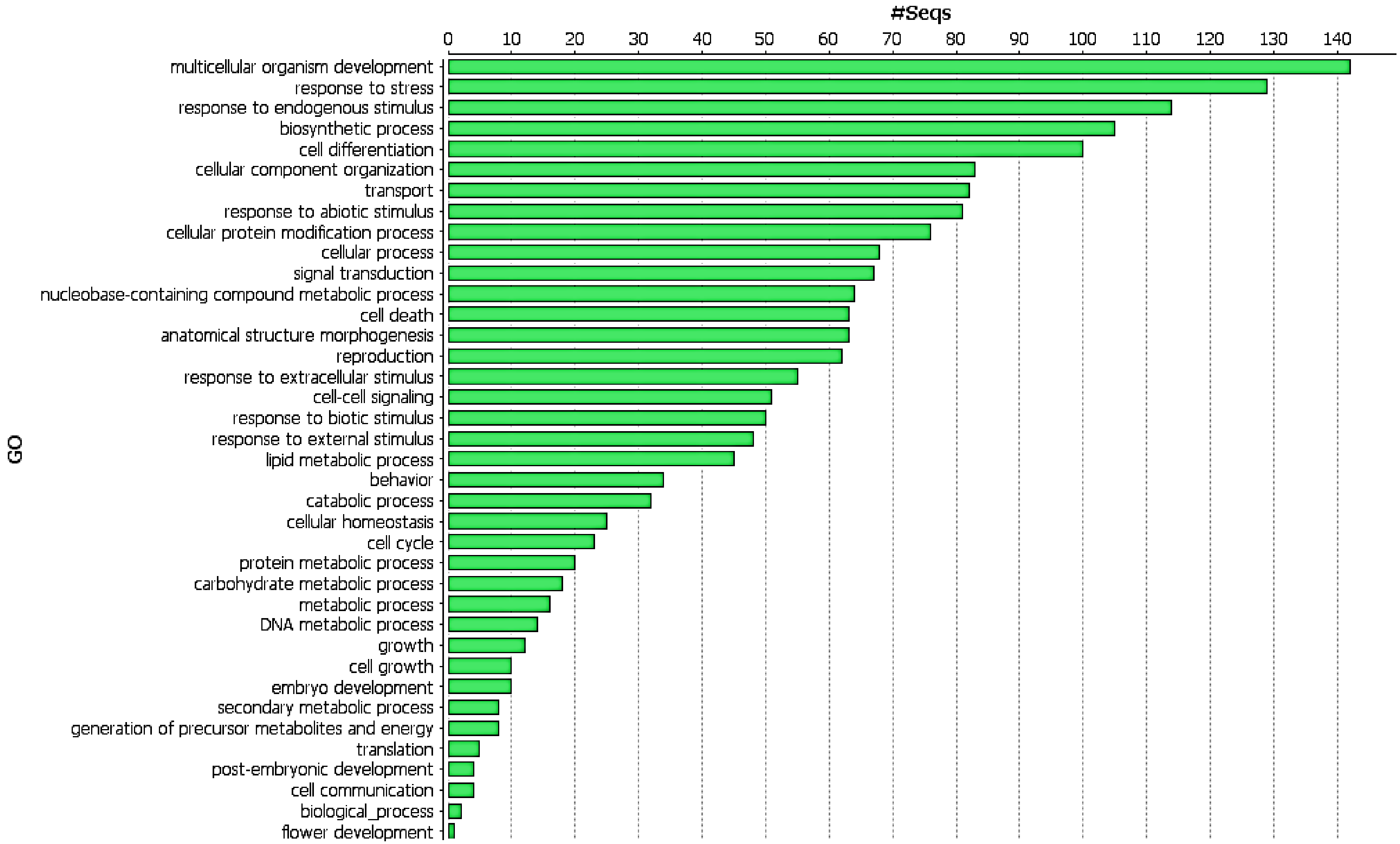

Supplementary Fig. 1. Gene ontology classification of top 20 sub-groups of biological processes.

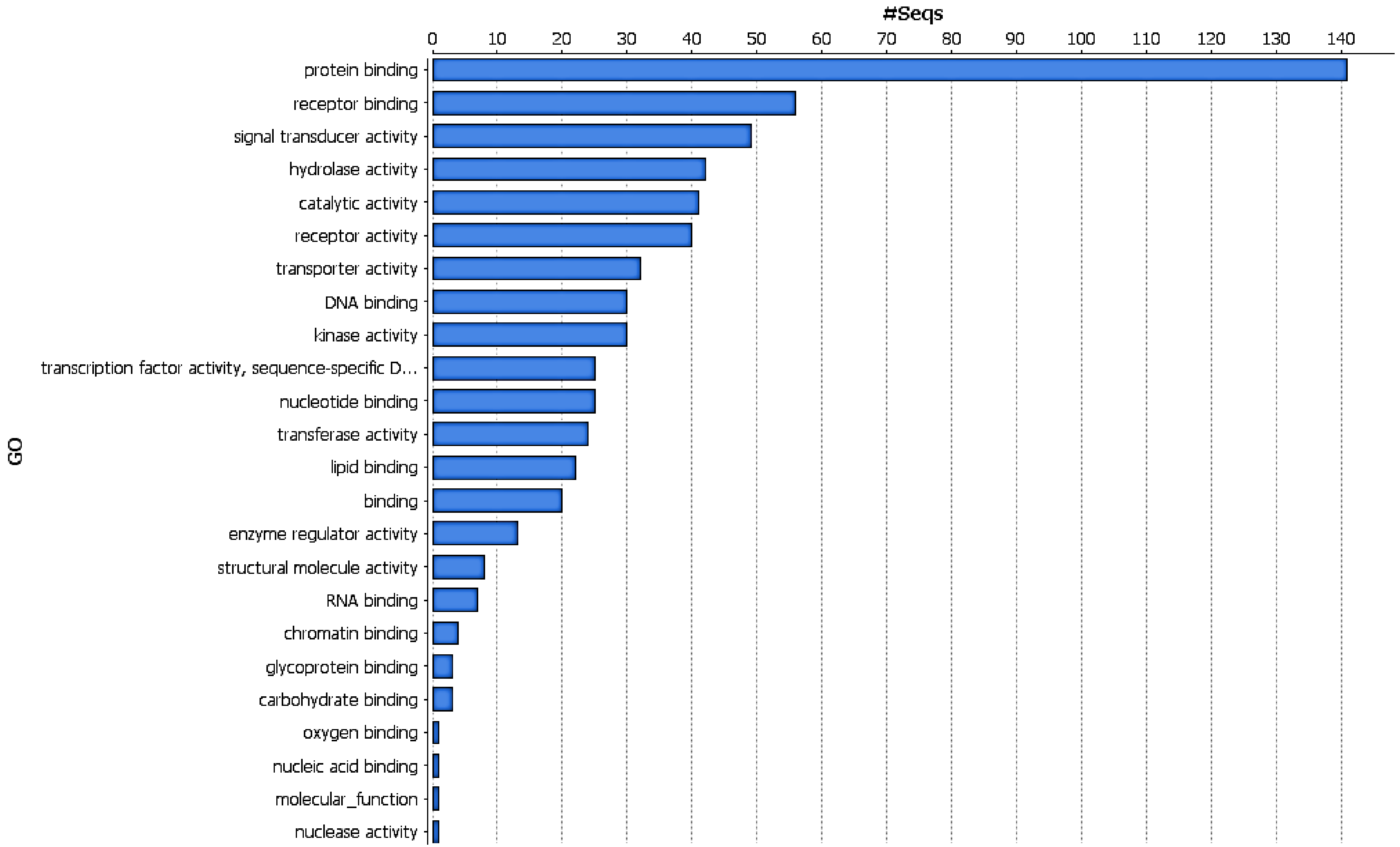

Supplementary Fig. 2. Gene ontology classification of top 20 sub-groups of molecular functions.

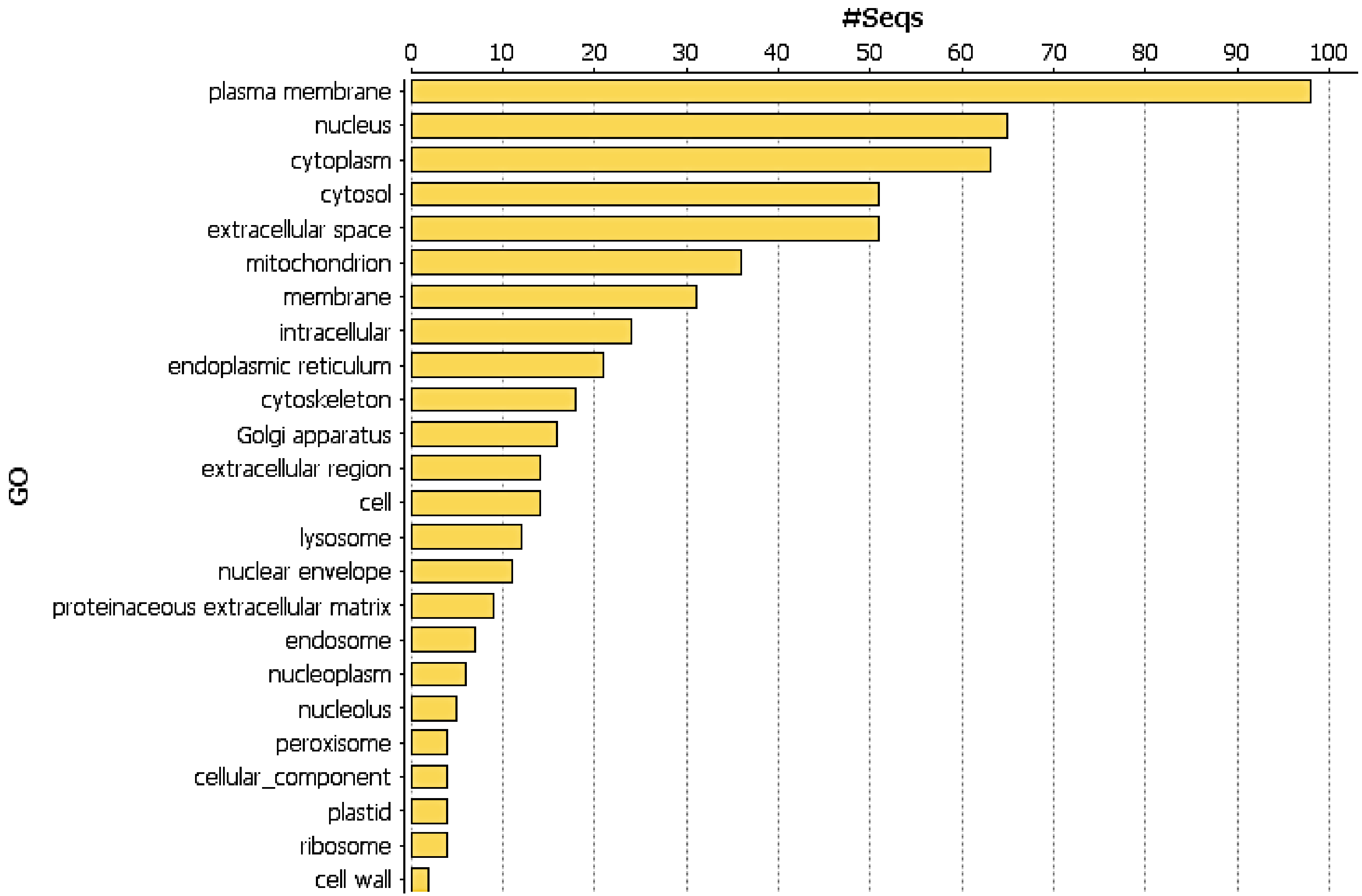

Supplementary Fig. 3. Gene ontology classification of top 20 sub-groups of cellular components.

Supplementary Table 1. List of putative QTL identified from KEGG pathways with GO terms in *C. sinensis* transcriptome

Nr	Trait	Locus	LG	Pos (cM)	LOD	PEV	E-Value	#GO	GO IDs	GO Name	Pathway	#Enzs in Pathway	Enzyme Codes
1	[a]TF1	5084595	2	4.5	3.2	5.7	3.7E-23	9	F:GO:0000166;<br>F:GO:0003824;<br>P:GO:0007275;<br>P:GO:0006629;<br>P:GO:0030154;<br>F:GO:0008289;<br>P:GO:0009058;<br>C:GO:0005622;<br>F:GO:0005215	F:nucleotide binding;<br>F:catalytic activity;<br>P:multicellular organism development;<br>P:lipid metabolic process;<br>P:cell differentiation;<br>F:lipid binding;<br>P:biosynthetic process;<br>C:intracellular;<br>F:transporter activity	Steroid degradation, Steroid hormone biosynthesis	11	EC:1.3.99.5 - 4-dehydrogenase (acceptor),<br>EC:1.1.1.145 - dehydrogenase,<br>EC:1.14.15.4 - 11beta-monooxygenase,<br>EC:1.14.15.5 - 18-monooxygenase,<br>EC:2.8.2.4 - sulfotransferase,<br>EC:2.1.1.6 - O-methyltransferase,<br>EC:1.14.14.1 - monooxygenase,<br>EC:1.1.1.62 - 17-dehydrogenase,<br>EC:1.1.1.239 - dehydrogenase (NAD+),<br>EC:1.1.1.145 - dehydrogenase,<br>EC:2.4.1.17 - 1-naphthol glucuronyltransferase
2	[a]TF2	5084595	2	4.5	4.1	7.3	3.7E-23	9	F:GO:0000166;<br>F:GO:0003824;<br>P:GO:0007275;<br>P:GO:0006629;<br>P:GO:0030154;<br>F:GO:0008289;<br>P:GO:0009058;<br>C:GO:0005622;<br>F:GO:0005215	F:nucleotide binding;<br>F:catalytic activity;<br>P:multicellular organism development;<br>P:lipid metabolic process;<br>P:cell differentiation;<br>F:lipid binding;<br>P:biosynthetic process;<br>C:intracellular;<br>F:transporter activity	Steroid degradation, Steroid hormone biosynthesis	11	EC:1.3.99.5 - 4-dehydrogenase (acceptor),<br>EC:1.1.1.145 - dehydrogenase,<br>EC:1.14.15.4 - 11beta-monooxygenase,<br>EC:1.14.15.5 - 18-monooxygenase,<br>EC:2.8.2.4 - sulfotransferase,<br>EC:2.1.1.6 - O-methyltransferase,<br>EC:1.14.14.1 - monooxygenase,<br>EC:1.1.1.62 - 17-dehydrogenase,<br>EC:1.1.1.239 - dehydrogenase (NAD+),<br>EC:1.1.1.145 - dehydrogenase,<br>EC:2.4.1.17 - 1-naphthol glucuronyltransferase
3	[a]EGC	5124128	2	7.7	3.3	5.6	2.2E-18	1	F:GO:0005198	F:structural molecule activity	-	-	-
4	[a]ECG	5098382	6	57	5.2	8.7	5.5E-19	8	F:GO:0003723;<br>F:GO:0005515;<br>P:GO:0007275;<br>P:GO:0009653;<br>C:GO:0005730;<br>P:GO:0006139;<br>C:GO:0005654;<br>F:GO:0016787	F:RNA binding;<br>F:protein binding;<br>P:multicellular organism development;<br>P:anatomical structure morphogenesis;<br>C:nucleolus;<br>P:nucleobase-containing compound metabolic	Purine metabolism	15	EC:4.6.1.1 - cyclase,<br>EC:4.6.1.2 - cyclase,<br>EC:2.7.4.6 - kinase,<br>EC:2.7.4.3 - kinase,<br>EC:3.6.1.3 - adenylpyrophosphatase,<br>EC:3.1.4.17 - phosphodiesterase,<br>EC:3.5.4.4 - deaminase,<br>EC:2.7.1.4- - kinase,<br>EC:3.5.4.6 - deaminase,

										process; C:nucleoplasm;<br>F:hydrolase activity			EC:3.1.4.35 - phosphodiesterase,<br>EC:2.7.1.2- - kinase,<br>EC:3.6.1.15 - phosphatase,<br>EC:5.4.2.2 - (alpha-D-glucose-1,6-bisphosphate-dependent),<br>EC:2.7.6.1 - diphosphokinase,<br>EC:2.4.2.8 - phosphoribosyltransferase
5	[a]TF1	5136045	6	69.6	4.3	7.7	5.5E-19	1	F:GO:0005515	F:protein binding	-	-	-
6	[a]CAFF	5111497	8	18.8	4.1	7.1	3.7E-23	4	P:GO:0008150;<br>F:GO:0005515;<br>C:GO:0005737;<br>C:GO:0005576	P:biological_process;<br>F:protein binding;<br>C:cytoplasm;<br>C:extracellular region	-	-	-
7	[a]CAT	5103784	13	50.6	3.8	6.1	5.5E-19	14	P:GO:0007275;<br>P:GO:0007165;<br>P:GO:0008152;<br>P:GO:0007267;<br>C:GO:0005886;<br>P:GO:0007049;<br>C:GO:0005635;<br>C:GO:0005856;<br>F:GO:0005515;<br>P:GO:0008219;<br>C:GO:0005737;<br>P:GO:0009719;<br>P:GO:0016043;<br>P:GO:0030154	P:multicellular organism development;<br>P:signal transduction;<br>P:metabolic process;<br>P:cell-cell signaling;<br>C:plasma membrane;<br>P:cell cycle;<br>C:nuclear envelope;<br>C:cytoskeleton;<br>F:protein binding;<br>P:cell death;<br>C:cytoplasm;<br>P:response to endogenous stimulus;<br>P:cellular component organization;<br>P:cell differentiation	-	-	-
8	[a]CAT	5122899	13	50.5	3.8	6	3.7E-23	5	F:GO:0005515;<br>P:GO:0007275;<br>P:GO:0030154;<br>C:GO:0005886;<br>F:GO:0005215	F:protein binding;<br>P:multicellular organism development;<br>P:cell differentiation;<br>C:plasma membrane;<br>F:transporter activity	-	-	-
9	[a]ECG	5123761	13	60.7	3.3	5.8	3.6E-20	2	F:GO:0003700;<br>C:GO:0005622	F:transcription factor activity, sequence-specific DNA binding;<br>C:intracellular	-	-	-
10	[a]BRT	5135810	13	26	2.8	5.8	1.3E-13	4	C:GO:0005623;<br>P:GO:0019538;	C:cell;	-	-	-

									F:GO:0005515;<br>F:GO:0016787	P:protein metabolic process;<br>F:protein binding;<br>F:hydrolase activity			
11	[a]CAFF	5054639	14	6.4	3.9	6.9	5.0E-10	12	C:GO:0005615;<br>P:GO:0009628;<br>P:GO:0008219;<br>P:GO:0007275;<br>C:GO:0005737;<br>P:GO:0009719;<br>P:GO:0007267;<br>P:GO:0016043;<br>P:GO:0030154;<br>P:GO:0009653;<br>F:GO:0005102;<br>P:GO:0006950	C:extracellular space;<br>P:response to abiotic stimulus;<br>P:cell death;<br>P:multicellular organism development;<br>C:cytoplasm;<br>P:response to endogenous stimulus;<br>P:cell-cell signaling;<br>P:cellular component organization;<br>P:cell differentiation;<br>P:anatomical structure morphogenesis;<br>F:receptor binding;<br>P:response to stress	-	-	-
12	[a]CAT	5132370	14	60.7	6.5	10.8	1.5E-25	26	P:GO:0007275;<br>P:GO:0009991;<br>P:GO:0006464;<br>F:GO:0008289;<br>P:GO:0009653;<br>P:GO:0009058;<br>P:GO:0009607;<br>P:GO:0009628;<br>F:GO:0003723;<br>P:GO:0008219;<br>P:GO:0007165;<br>P:GO:0006259;<br>F:GO:0005102;<br>P:GO:0006810;<br>P:GO:0006950;<br>P:GO:0000003;<br>P:GO:0007049;<br>P:GO:0040007;<br>C:GO:0005634;<br>F:GO:0003677;	P:multicellular organism development;<br>P:response to extracellular stimulus;<br>P:cellular protein modification process;<br>F:lipid binding;<br>P:anatomical structure morphogenesis;<br>P:biosynthetic process;<br>P:response to biotic stimulus;<br>P:response to abiotic stimulus;<br>F:RNA binding;<br>P:cell death;<br>P:signal transduction;<br>P:DNA metabolic process;	-	-	-

									C:GO:0005615;<br>C:GO:0005737;<br>P:GO:0009719;<br>P:GO:0016043;<br>P:GO:0030154;<br>F:GO:0016740	F:receptor binding;<br>P:transport;<br>P:response to stress;<br>P:reproduction;<br>P:cell cycle;<br>P:growth;<br>C:nucleus;<br>F:DNA binding;<br>C:extracellular space;<br>C:cytoplasm;<br>P:response to endogenous stimulus;<br>P:cellular component organization;<br>P:cell differentiation;<br>F:transferase activity			
13	[a]EGC	5132370	14	60.7	3.7	6.4	1.5E-25	26	P:GO:0007275;<br>P:GO:0009991;<br>P:GO:0006464;<br>F:GO:0008289;<br>P:GO:0009653;<br>P:GO:0009058;<br>P:GO:0009607;<br>P:GO:0009628;<br>F:GO:0003723;<br>P:GO:0008219;<br>P:GO:0007165;<br>P:GO:0006259;<br>F:GO:0005102;<br>P:GO:0006810;<br>P:GO:0006950;<br>P:GO:0000003;<br>P:GO:0007049;<br>P:GO:0040007;<br>C:GO:0005634;<br>F:GO:0003677;<br>C:GO:0005615;<br>C:GO:0005737;<br>P:GO:0009719;<br>P:GO:0016043;	P:multicellular organism development;<br>P:response to extracellular stimulus;<br>P:cellular protein modification process;<br>F:lipid binding;<br>P:anatomical structure morphogenesis;<br>P:biosynthetic process;<br>P:response to biotic stimulus;<br>P:response to abiotic stimulus;<br>F:RNA binding;<br>P:cell death;<br>P:signal transduction;<br>P:DNA metabolic process;<br>F:receptor binding;<br>P:transport;<br>P:response to stress;<br>P:reproduction;<br>P:cell cycle;<br>P:growth;	-	-	-

									P:GO:0030154; F:GO:0016740	C:nucleus; F:DNA binding; C:extracellular space; C:cytoplasm; P:response to endogenous stimulus; P:cellular component organization; P:cell differentiation; F:transferase activity			
14	[a]EGCG	5114089	15	32.1	4	6.8	8.0E-12	5	C:GO:0005634; F:GO:0003700; P:GO:0007275; P:GO:0009058; P:GO:0006139	C:nucleus; F:transcription factor activity, sequence-specific DNA binding; P:multicellular organism development; P:biosynthetic process; P:nucleobase-containing compound metabolic process	-	-	-
15	[b]TF1	5084595	2	4.5	3.2	5.7	3.7E-23	9	F:GO:0000166; F:GO:0003824; P:GO:0007275; P:GO:0006629; P:GO:0030154; F:GO:0008289; P:GO:0009058; C:GO:0005622; F:GO:0005215	F:nucleotide binding; F:catalytic activity; P:multicellular organism development; P:lipid metabolic process; P:cell differentiation; F:lipid binding; P:biosynthetic process; C:intracellular; F:transporter activity	Steroid degradation, Steroid hormone biosynthesis	11	EC:1.3.99.5 - 4-dehydrogenase (acceptor), EC:1.1.1.145 - dehydrogenase, EC:1.14.15.4 - 11beta-monooxygenase, EC:1.14.15.5 - 18-monooxygenase, EC:2.8.2.4 - sulfotransferase, EC:2.1.1.6 - O-methyltransferase, EC:1.14.14.1 - monooxygenase, EC:1.1.1.62 - 17-dehydrogenase, EC:1.1.1.239 - dehydrogenase (NAD+), EC:1.1.1.145 - dehydrogenase, EC:2.4.1.17 - 1-naphthol glucuronyltransferase
16	[b]TF2	5084595	2	4.5	4.1	7.3	3.7E-23	9	F:GO:0000166; F:GO:0003824; P:GO:0007275; P:GO:0006629; P:GO:0030154; F:GO:0008289; P:GO:0009058;	F:nucleotide binding; F:catalytic activity; P:multicellular organism development; P:lipid metabolic process; P:cell differentiation; F:lipid binding;	Steroid degradation, Steroid hormone biosynthesis	11	EC:1.3.99.5 - 4-dehydrogenase (acceptor), EC:1.1.1.145 - dehydrogenase, EC:1.14.15.4 - 11beta-monooxygenase, EC:1.14.15.5 - 18-monooxygenase, EC:2.8.2.4 - sulfotransferase, EC:2.1.1.6 - O-methyltransferase,

									C:GO:0005622;<br>F:GO:0005215	P:biosynthetic process;<br>C:intracellular;<br>F:transporter activity			EC:1.14.14.1 - monooxygenase,<br>EC:1.1.1.62 - 17-dehydrogenase,<br>EC:1.1.1.239 - dehydrogenase (NAD+),<br>EC:1.1.1.145 - dehydrogenase,<br>EC:2.4.1.17 - 1-naphthol glucuronyltransferase
17	[b]EGC	5124128	2	7.7	3.3	5.6	2.2E-18	1	F:GO:0005198	F:structural molecule activity	-	-	-
18	[b]ECG	5098382	6	57	5.2	8.7	5.5E-19	8	F:GO:0003723;<br>F:GO:0005515;<br>P:GO:0007275;<br>P:GO:0009653;<br>C:GO:0005730;<br>P:GO:0006139;<br>C:GO:0005654;<br>F:GO:0016787	F:RNA binding;<br>F:protein binding;<br>P:multicellular organism development;<br>P:anatomical structure morphogenesis;<br>C:nucleolus;<br>P:nucleobase-containing compound metabolic process;<br>C:nucleoplasm;<br>F:hydrolase activity	Purine metabolism	15	EC:4.6.1.1 - cyclase,<br>EC:4.6.1.2 - cyclase,<br>EC:2.7.4.6 - kinase,<br>EC:2.7.4.3 - kinase,<br>EC:3.6.1.3 - adenylpyrophosphatase,<br>EC:3.1.4.17 - phosphodiesterase,<br>EC:3.5.4.4 - deaminase,<br>EC:2.7.1.4- - kinase,<br>EC:3.5.4.6 - deaminase,<br>EC:3.1.4.35 - phosphodiesterase,<br>EC:2.7.1.2- - kinase,<br>EC:3.6.1.15 - phosphatase,<br>EC:5.4.2.2 - (alpha-D-glucose-1,6-bisphosphate-dependent),<br>EC:2.7.6.1 - diphosphokinase,<br>EC:2.4.2.8 - phosphoribosyltransferase
19	[b]CAT	5132370	14	60.7	6.5	10.8	1.5E-25	26	P:GO:0007275;<br>P:GO:0009991;<br>P:GO:0006464;<br>F:GO:0008289;<br>P:GO:0009653;<br>P:GO:0009058;<br>P:GO:0009607;<br>P:GO:0009628;<br>F:GO:0003723;<br>P:GO:0008219;<br>P:GO:0007165;<br>P:GO:0006259;<br>F:GO:0005102;<br>P:GO:0006810;<br>P:GO:0006950;<br>P:GO:0000003;<br>P:GO:0007049;	P:multicellular organism development;<br>P:response to extracellular stimulus;<br>P:cellular protein modification process;<br>F:lipid binding;<br>P:anatomical structure morphogenesis;<br>P:biosynthetic process;<br>P:response to biotic stimulus;<br>P:response to abiotic stimulus;<br>F:RNA binding;<br>P:cell death;	-	-	-

									P:GO:0040007; C:GO:0005634; F:GO:0003677; C:GO:0005615; C:GO:0005737; P:GO:0009719; P:GO:0016043; P:GO:0030154; F:GO:0016740	P:signal transduction; P:DNA metabolic process; F:receptor binding; P:transport; P:response to stress; P:reproduction; P:cell cycle; P:growth; C:nucleus; F:DNA binding; C:extracellular space; C:cytoplasm; P:response to endogenous stimulus; P:cellular component organization; P:cell differentiation; F:transferase activity			
20	[b]EGCG	5114089	15	32.1	4	6.8	8.0E-12	5	C:GO:0005634; F:GO:0003700; P:GO:0007275; P:GO:0009058; P:GO:0006139	C:nucleus; F:transcription factor activity, sequence-specific DNA binding; P:multicellular organism development; P:biosynthetic process; P:nucleobase-containing compound metabolic process	-	-	-

[a] Putative QTL identified in Interval Mapping using Joinmap software v4.1.

[b] Putative QTL identified in Multiple QTL Model Mapping using Joinmap software v4.1

P (biological process); F (molecular function); C (cellular component)

(-) Not found in KEGG pathway

Supplementary Table 2. List of unigenes from primary and secondary metabolic pathways in 15 LGs of *C. sinensis* transcriptome

Nr	Pathway	#Enzs in Pathway	Enzyme	#Unigene	Unigene ID
1	Alanine, aspartate and glutamate metabolism	4	EC:4.1.1.15 - decarboxylase, EC:6.3.4.16 - synthase (ammonia),	7	5134557, 5082783, 5134557, 5082783, 5134840, 5098250, 5125548

			EC:1.4.1.2 - dehydrogenase, EC:2.6.1.44 - transaminase		
2	Amino sugar and nucleotide sugar metabolism	3	EC:5.4.2.2 - (alpha-D-glucose-1,6-bisphosphate-dependent), EC:2.7.1.2 - glucokinase (phosphorylating), EC:2.7.1.1 - hexokinase type IV glucokinase	4	5122964, 5077455, 5134772, 5134772
3	Arginine and proline metabolism	4	EC:4.1.1.50 - decarboxylase, EC:1.14.13.39 - synthase (NADPH), EC:3.5.3.1 - arginine amidinase, EC:1.2.1.3 - dehydrogenase (NAD+)	5	5124570, 5133545, 5124506, 5109744, 5123629
4	Arginine biosynthesis	6	EC:1.14.13.39 - synthase (NADPH), EC:3.5.3.1 - arginine amidinase, EC:6.3.4.16 - synthase (ammonia), EC:2.1.3.3 - carbamoyltransferase, EC:1.4.1.2 - dehydrogenase, EC:3.5.1.14 - acid amidohydrolase	8	5133545, 5124506, 5109744, 5134557, 5082783, 5098949, 5134840, 5080820
5	Ascorbate and aldarate metabolism	2	EC:2.4.1.17 - 1-naphthol glucuronyltransferase, EC:1.2.1.3 - dehydrogenase (NAD+)	3	5075058, 5084636, 5123629
6	Carbon fixation in photosynthetic organisms	2	EC:4.1.2.13 - aldolase, EC:1.1.1.37 - dehydrogenase	5	5106931, 5084452, 5133604, 5124439, 5132470
7	Cysteine and methionine metabolism	5	EC:4.1.1.50 - decarboxylase, EC:1.1.1.37 - dehydrogenase, EC:4.2.1.22 - beta-synthase, EC:1.1.1.27 - dehydrogenase, EC:2.6.1.44 - transaminase	9	5124570, 5133604, 5124439, 5132470, 5112205, 5123470, 5133941, 5098250, 5125548
8	Flavone and flavonol biosynthesis	1	EC:3.2.1.31 - beta-glucuronide glucuronohydrolase glucuronidase	2	5072939, 5068701
9	Fructose and mannose metabolism	4	EC:4.1.2.13 - aldolase, EC:3.2.1.78 - endo-1,4-beta-mannosidase, EC:1.1.1.21 - reductase, EC:2.7.1.1 - hexokinase type IV glucokinase	6	5106931, 5084452, 5059558, 5127501, 5123251, 5134772
10	Glycerolipid metabolism	5	EC:2.7.1.107 - kinase (ATP), EC:1.1.1.2 - dehydrogenase (NADP+),	6	5137030, 5123251, 5123251, 5134984, 5065613, 5123629

			EC:1.1.1.21 - reductase,<br>EC:3.1.1.3 - lipase, EC:1.2.1.3 - dehydrogenase (NAD+)		
11	Glycine, serine and threonine metabolism	3	EC:4.2.1.22 - beta-synthase,<br>EC:2.6.1.51 - transaminase,<br>EC:2.6.1.44 - transaminase	5	5112205, 5098250, 5125548, 5098250, 5125548
12	Glyoxylate and dicarboxylate metabolism	3	EC:6.4.1.3 - carboxylase,<br>EC:2.3.1.9 - C-acetyltransferase,<br>EC:1.1.1.37 - dehydrogenase	5	5122912, 5124564, 5133604, 5124439, 5132470
13	Histidine metabolism	1	EC:1.2.1.3 - dehydrogenase (NAD+)	1	5123629
	mTOR signaling pathway	1	EC:2.7.11.24 - protein kinase	1	5132197
	Nitrogen metabolism	2	EC:6.3.4.16 - synthase (ammonia),<br>EC:1.4.1.2 - dehydrogenase	3	5134557, 5082783, 5134840
14	Other glycan degradation	2	EC:3.2.1.25 - mannanase,<br>EC:3.2.1.18 - neuraminidase	3	5059558, 5127501, 5055477
15	Pentose and glucuronate interconversions	4	EC:1.1.1.2 - dehydrogenase (NADP+),<br>EC:3.2.1.31 - beta-glucuronide glucuronohydrolase glucuronidase,<br>EC:1.1.1.21 - reductase,<br>EC:2.4.1.17 - 1-naphthol glucuronyltransferase	6	5123251, 5072939, 5068701, 5123251, 5075058, 5084636
16	Phosphatidylinositol signaling system	2	EC:2.7.1.107 - kinase (ATP),<br>EC:3.1.4.11 - phospholipase C	1	5137030, 5122970
17	Porphyrin and chlorophyll metabolism	3	EC:4.2.1.24 - synthase,<br>EC:3.2.1.31 - beta-glucuronide glucuronohydrolase glucuronidase,<br>EC:2.4.1.17 - 1-naphthol glucuronyltransferase	3	5132295, 5072939, 5068701, 5075058, 5084636
18	Purine metabolism	6	EC:3.6.1.15 - phosphatase,<br>EC:2.7.4.3 - kinase,<br>EC:3.6.1.3 - adenylpyrophosphatase,<br>EC:5.4.2.2 - (alpha-D-glucose-1,6-bisphosphate-dependent),<br>EC:3.1.4.17 - phosphodiesterase,<br>EC:3.5.4.4 - deaminase	22	5123251, 5090210, 5133369, 5125437, 5082614, 5118156, 5105900, 5078215, 5134705, 5137364, 5113142, 5136410, 5125437, 5090210, 5133369, 5105900, 5078215, 5122964, 5077455, 5123174, 5075058, 5098382

19	Pyruvate metabolism	5	EC:2.3.1.9 - C-acetyltransferase, EC:1.1.1.37 - dehydrogenase, EC:4.4.1.5 - lyase, EC:1.1.1.27 - dehydrogenase, EC:1.2.1.3 - dehydrogenase (NAD+)	8	5124564, 5133604, 5124439, 5132470, 5136472, 5123470, 5133941, 5123629
20	Terpenoid backbone biosynthesis	3	EC:2.3.3.10 - synthase, EC:2.3.1.9 - C-acetyltransferase, EC:2.5.1.29 - diphosphate synthase	4	5125140, 5124564, 5096779, 5072048
21	Thiamine metabolism	2	EC:3.6.1.15 - phosphatase, EC:2.7.4.3 - kinase	12	5123251, 5090210, 5133369, 5125437, 5082614, 5118156, 5105900, 5078215, 5134705, 5137364, 5113142, 5136410
22	Tryptophan metabolism	2	EC:2.3.1.9 - C-acetyltransferase, EC:1.2.1.3 - dehydrogenase (NAD+)	2	5124564, 5123629
23	Tyrosine metabolism	1	EC:2.1.1.6 - O-methyltransferase	2	5059558, 5081364
24	Ubiquinone and other terpenoid-quinone biosynthesis	1	EC:1.6.5.2 - dehydrogenase (quinone)	1	5107213
25	Valine, leucine and isoleucine degradation	5	EC:2.3.3.10 - synthase, EC:6.4.1.3 - carboxylase, EC:2.3.1.9 - C-acetyltransferase, EC:2.3.1.16 - C-acyltransferase, EC:1.2.1.3 - dehydrogenase (NAD+)	5	5125140, 5122912, 5124564, 5124564, 5123629

24
